# Integrative transcriptomics reveals an ECM/PNN-high astrocyte state and cross-tissue biomarkers in major depressive disorder

**DOI:** 10.3389/fnmol.2026.1995597

**Published:** 2026-10-23

**Authors:** Yifan Wang, Chenrui Liang, Jiang Fu, Huating Liu, Yiting Lei, Zijing Liu, Shuhang He, Xiyan Jiang, Donghang Ren, Enqiang Dong, Lin Yang, Lixia Wang

**Affiliations:** 1 The First Affiliated Hospital of Dali University, Dali, Yunnan, China; 2 Dali University, Dali, Yunnan, China

**Keywords:** astrocytes, biomarkers, extracellular matrix, major depressive disorder, perineuronal net

## Abstract

**Background:**

Major depressive disorder (MDD) is associated with impaired neural plasticity, while extracellular matrix (ECM) and perineuronal nets (PNNs) are important regulators of synaptic stability and extracellular homeostasis. However, the cellular states and molecular signatures associated with ECM/PNN alterations in MDD remain poorly understood.

**Methods:**

We integrated bulk and single-cell transcriptomic datasets to characterize ECM/PNN-associated cellular states in MDD. Astrocyte populations were evaluated using multiple ECM/PNN scoring approaches, and disease-associated transcriptional changes were integrated with astrocyte co-expression networks to identify candidate molecular signatures. Functional enrichment, cell–cell communication, pseudotime, and in silico perturbation analyses were further used to investigate their biological context. Finally, corticosterone (CORT)-treated HT22 cells were used as a glucocorticoid-induced neuronal stress paradigm to assess stress-responsive expression of the identified genes.

**Results:**

Among astrocyte clusters, Cluster2 exhibited overall high ECM and PNN signature scores. Integration of Cluster2-associated co-expression modules with MDD-related differentially expressed genes identified 28 overlapping genes, from which ACSL6, FIP1L1, and ZNF709 were prioritized as cross-tissue candidate signatures showing consistent expression trends in brain and peripheral blood datasets. ACSL6 and ZNF709 were downregulated, whereas FIP1L1 was upregulated in MDD. Functional analyses implicated the three genes in metabolic and amino-acid-related processes, while FIP1L1 showed additional associations with cell adhesion, cytoskeletal organization, and synaptic signaling. Cell communication and pseudotime analyses further suggested altered intercellular signaling and increasing ECM/PNN activity across inferred astrocyte state transitions. In CORT-treated HT22 cells, ACSL6 and ZNF709 were significantly decreased, whereas FIP1L1 was increased, consistent with the transcriptomic observations.

**Conclusion:**

These findings identify an ECM/PNN-high astrocyte state and three associated cross-tissue molecular signatures in MDD. Among them, FIP1L1 emerged as a functionally prioritized candidate linked to adhesion- and synaptic-plasticity-related programs. The stress-responsive expression of these genes provides additional experimental support for their relevance to glucocorticoid-associated neuronal stress and supports an astrocyte-centered ECM/PNN working model for further mechanistic investigation.

## 1 Introduction

Major depressive disorder (MDD) is characterized by persistently depressed mood and diminished interest or loss of pleasure (anhedonia) as core symptoms, and is often accompanied by cognitive impairment, changes in sleep and appetite, psychomotor abnormalities, and an increased risk of suicide (Belmaker and Agam, 2008). Patients with MDD show marked disease heterogeneity (Kennedy and Ceniti, 2018). The pathogenesis of MDD involves multiple pathological processes, including dysregulation of monoaminergic signaling, hypothalamic–pituitary–adrenal axis dysfunction, neuroimmune dysregulation, glial cell dysfunction, and impaired synaptic plasticity (Belmaker and Agam, 2008). Currently, the main treatments for MDD include antidepressant medications, psychotherapy, and electroconvulsive therapy (Fava and Kendler, 2000). However, current treatments do not guarantee sustained remission in all patients, and in some individuals, treatment outcomes remain unsatisfactory even after multiple adjustments. A large-scale, multicenter sequential treatment study of depression showed that with each additional treatment step, the likelihood of achieving remission gradually decreased, while the risk of relapse during follow-up correspondingly increased (Rush et al., 2006). Moreover, there is still a lack of biomarkers of MDD that are robust enough for routine clinical use and can objectively reflect the underlying pathological changes (Kennedy and Ceniti, 2018). Therefore, identifying molecular biomarkers are necessary to predict treatment response.

Impaired neural plasticity is considered an important underlying basis for the onset and progression of MDD, and the extracellular matrix (ECM) is a key component in regulating neural plasticity. The ECM is mainly composed of collagens, glycoproteins, proteoglycans, and related regulatory molecules. It not only provides structural support but also participates in cell adhesion, migration, and signal transduction through interactions with cell-surface receptors and dynamic remodeling (Theocharis et al., 2016). The perineuronal net (PNN) is a highly specialized ECM structure in the central nervous system that primarily surrounds a subset of inhibitory interneurons and plays an important role in stabilizing synaptic connections, regulating critical-period plasticity during development, and maintaining the excitation/inhibition balance (Miao et al., 2014; Fawcett et al., 2019). Previous studies have shown that ECM/PNN abnormalities are linked to impaired synaptic plasticity in various neuropsychiatric disorders (Testa et al., 2019). In MDD-related research, chronic stress can induce ECM/PNN remodeling in the brain, whereas interventions targeting PNN structure can improve synaptic function and alleviate cognitive impairments in animal models of depression (Riga et al., 2017; Blanco and Conant, 2021). In addition, human genetic studies have found that ECM degradation–related pathways are associated with MDD susceptibility (Zailani et al., 2025), and recent animal studies have suggested that PNN formation mediated by Semaphorin 3A may be involved in maintaining depression-like behavior (Mei et al., 2026). Therefore, screening candidate ECM- and PNNs-related biomarkers may provide new clues for understanding the neural plasticity abnormalities underlying MDD.

Given their important roles in extracellular homeostasis and ECM/PNN remodeling, astrocytes were selected as the primary cellular focus, although other glial cell types may also contribute to these processes (Faissner et al., 2010; Simard et al., 2018). We hypothesized that MDD may involve an astrocyte state characterized by altered ECM/PNN signatures. To investigate this possibility, we integrated bulk transcriptomic data, which captured MDD-associated tissue-level expression changes, with scRNA-seq data, which enabled characterization of cell-state-specific ECM/PNN features. We then got some idea of what those biomarkers might be doing functionally. Separately, we built a glucocorticoid‑induced neuronal stress model *in vitro*, to see whether these biomarkers would show transcriptional changes under stress. Ultimately, our work is meant to generate testable hypotheses for future mechanistic studies—especially for those interested in how ECM‑PNN contributes to the neuropathology of depression.

## 2 Materials and methods

### 2.1 Data source

MDD-related datasets were downloaded from the Gene Expression Omnibus (GEO^1^) database. Training set (GSE54564, platform: GPL6947) comprised human brain amygdala samples with 21 MDD and 21 control samples. Validation set (GSE76826, platform: GPL17077) consisted of human peripheral blood samples with 20 MDD and 12 control samples. Single-cell RNA sequencing (scRNA-seq) datasets, GSE144136 (platform: GPL20301, frontal cortex, 17 MDD and 17 control samples) and GSE213982 (platform: GPL24676 and GPL28038, frontal cortex, 20 MDD and 18 control samples), were incorporated and pooled for downstream analyses.

Moreover, PNNs-related genes (PNNGs) were retrieved from the Msigdb database^2^ under the entry GOCC_PERINEURONAL_NET, encompassing 10 genes: ACAN, BCAN, HAPLN1, HAPLN2, HAPLN3, HAPLN4, NCAN, PTPRZ1, TNR, and VCAN. ECM-related genes (ECMGs) were obtained from the MatrisomeDB 2.0 database^3^ with the following filtering criteria: categories included Collagens, ECM Glycoproteins, Proteoglycans, and ECM Regulators; species restricted to Human; and tissue restricted to Blood vessel.

### 2.2 Single-cell analysis

In scRNA-seq dataset, quality control (QC) was performed through the Seurat package (version 5.1.0; Hao et al., 2021) with following eliminated thresholds: (1) number of genes per cell (nFeature_RNA) ≤ 200 or ≥ 6,000; (2) number of counts per cell (nCount_RNA) ≤ 200 or ≥ 40,000; (3) genes covered by fewer than three cells; and (4) cells with hemoglobin gene proportion > 1%. After normalization, vst method was utilized to extract top 2,000 highly variable genes (HVGs). To reduce the influence of doublets, potential doublets were identified and removed by the scrublet package (version 0.2.3) (Wolock et al., 2019) from Python. Subsequently, scRNA-seq data were scaled via ScaleData function, and top 30 principal components (PCs) were selected using JackStrawPlot function for further analysis. After performing principal component analysis (PCA) dimensionality reduction, FindNeighbors and FindClusters functions in the Seurat package were utilized for unsupervised cluster analysis to identify cell clusters with the resolution = 0.1. The results were visualized by uniform manifold approximation and projection (UMAP) method. According to marker genes in published literature (Nagy et al., 2020; Lutz et al., 2021; Maitra et al., 2023), cell types were annotated, and their proportion in diverse samples was compared.

Subsequently, target cell type (associated with PNNs and ECM) was extracted and reclustered following the workflow described above with a clustering resolution of 0.3. Cell cycle phases were predicted by the scran package (version 1.26.2; Lun et al., 2016). The 50 Hallmark pathways scores across each cluster of the target cell type were calculated using the GSVA package (version 2.2.0; Hänzelmann et al., 2013). The FindAllMarkers function was utilized to identify HVGs for each cluster (adj. *P* < 0.05), followed by functional enrichment analysis on HVGs of all reclustered clusters.

Moreover, five algorithms (AUCell, UCell, singscore, ssGSEA, and AddModuleScore) integrated in the irGSEA package (version 3.3.2; Fan et al., 2024) were applied to calculate the PNNs and ECM scores of cell clusters of the target cell type. Notably, cell clusters with high average scores of PNNs and ECM signatures were recognized as key cells.

### 2.3 High-dimensional weighted gene co-expression network analysis (hdWGCNA)

HdWGCNA was employed to identify genes associated with PNNs and ECM through the hdWGCNA package (version 0.3.03; Morabito et al., 2021). The optimal soft threshold was determined by calculating network topological metrics using the TestSoftPowers function, following construction and configuration of the expression matrix from the Seurat package via the SetupForWGCNA and SetDatExpr functions. The topological overlap matrix was calculated based on gene expression similarity, and hierarchical clustering was performed to analyze correlations between gene modules in the co-expression network. The network hierarchy was visualized by dendrogram. Finally, module eigengenes were extracted for each module, and the correlation between each gene and the corresponding module eigengene (gene connectivity, kME) was calculated. Using PNNs and ECM scores as traits, Pearson analysis was conducted between traits and modules. Those modules with cor > 0.3 were determined as key modules, and genes in them were key module genes.

### 2.4 Differential expression analysis

In training set, differential expression analysis was performed between control and MDD samples using the limma package (version 3.54.2; Ritchie et al., 2015) to identify differentially expressed genes (DEGs; *P* < 0.05). Volcano plots and heat maps were drafted to visualize these DEGs utilizing the ggplot2 (version 3.5.1; Ito and Murphy, 2013) and the pheatmap (version 1.0.12; Zhang et al., 2023) packages, respectively.

### 2.5 Identification of intersection genes and functional enrichment

The key module genes were intersected with DEGs to obtain intersection genes. Furthermore, Gene ontology (GO) and Kyoto Encyclopedia of Genes and Genomes (KEGG) enrichment analyses were performed on intersection genes (*P* < 0.05) via the clusterProfiler package (version 4.6.2; Yu et al., 2012).

### 2.6 Identification of biomarkers and their expressions

Two machine learning algorithms, least absolute shrinkage and selection operator (LASSO) and support vector machine-recursive feature elimination (SVM-RFE), were employed for the identification of candidate feature genes. LASSO regression analysis was conducted using the glmnet package (version 4.1-8; Friedman et al., 2010) with 10-fold cross-validation to select informative genes and optimize model parameters. SVM-RFE was carried out using the e1071 package (version 1.7-14; Shi et al., 2023) to rank genes according to their contribution to classification. The feature subset with the lowest cross-validation error was retained. The intersection of feature genes identified by both algorithms was considered as common candidate genes for subsequent analyses. Additionally, those genes with the significant difference and consistent expression trends were identified as biomarkers in training set and validation set (*P* < 0.05). In addition, violin plots were generated via the ggplot2 package to analyze the expression heterogeneity of biomarkers in annotated cell types.

### 2.7 Gene set enrichment analysis (GSEA)

Spearman analysis was performed between biomarkers and all genes in training set by the psych package (version 2.4.3; Robles-Jimenez et al., 2021), and their correlation coefficients were ranked thresholds. According to KEGG and Hallmark background gene set downloading from the msigdbr package (version 24.1.0)^4^, GSEA was conducted via the clusterProfiler package (|NES| > 1 and *P* < 0.05).

### 2.8 Drug prediction and molecular docking

Details for drug‑target prediction and molecular docking analyses are provided in Supplementary material.

### 2.9 Cell communication, pseudotime analysis and virtual perturbation

Cell communication analysis was performed between key cells and other annotated cell types in control and MDD groups. Core pathways across groups were visualized via heatmaps. Subsequently, pseudotime analysis of target cell type was conducted using the monocle package (version 2.26.0; Trapnell et al., 2014). Notably, the distribution of PNNs, ECM scores and biomarker expressions along pseudotime branches was visualized.

Geneformer was selected because it enables cell-state-aware representation of single-cell transcriptomic data and supports in silico perturbation analysis for hypothesis-generating assessment of candidate gene function. Specifically, the 12-layer pretrained Geneformer model [gf-12L-30M-i2048 (version 0.0.1; Theodoris et al., 2023)] was fine-tuned on key cells from MDD and control groups. Model training was implemented via the Classifier.validate function with the AdamW optimizer, an initial learning rate of 1 × 10^–4^, a linear scheduler with 500 warm-up steps, 10 training epochs, a batch size of 12, a maximum input of 2,048 genes, and a fixed random seed of 73. The first two layers of the model were frozen, computation was accelerated by an NVIDIA GeForce RTX 3080 GPU, and model outputs were visualized. Subsequently, virtual perturbation analysis was performed on key cells with the fine-tuned Geneformer model. Gene knockout simulation was conducted with the InSilicoPerturber function under parameters emb_mode = "cell" and forward_batch_size = 256. Perturbation outputs were summarized using InSilicoPerturberStats in aggregate_gene_shifts mode. Cosine_sim_mean quantified embedding similarity before and after perturbation, and N_Detections indicated the number of input cells containing the relevant gene or gene combination. After virtual knockout of biomarkers, the top 100 perturbed genes with the minimum Cosine_sim_mean and N_Detections ≥ 100 were screened. Finally, GO and KEGG enrichment analyses were performed on perturbed genes (*P* < 0.05).

### 2.10 Cell culture and model construction

Mouse hippocampal HT22 cells (Immocell, IM-M038) were cultured in high-glucose DMEM medium supplemented with 10% FBS and 1% PS at 37 °C with 5% CO_2_. Cells were routinely passaged at a ratio of 1:2-1:4 upon reaching 80% confluence. For experiments, logarithmically growing cells were seeded onto culture plates and incubated until 70% confluence prior to intervention. To establish an *in vitro* glucocorticoid‑induced neuronal stress paradigm, HT22 cells were treated with 20 μM corticosterone (CORT) for 24 h, while cells incubated with routine complete medium served as the control group.

### 2.11 Cell counting kit-8 (CCK-8)

Logarithmically growing HT22 cells were seeded into a 96 well plate. After cell adhesion, CORT with various concentrations (0, 20, 50, 100, 150, 200, 250, and 300 μM) were added in each well. Following the culture of 24 h, 10 μL CCK-8 solution (Servicebio, G1613-5ML) were added, and the OD value in each well was detected using a microplate reader.

### 2.12 RT-qPCR

Total RNA was isolated using FastPure Complex Tissue/Cell Total RNA Isolation Kit (Vazyme, RC113-01) and quantified by a Nanodrop-500 (Allsheng, Nano500). RNA was then reverse-transcribed into cDNA through ABScript III RT Master Mix for RT-qPCR with gDNA Remover (Abclonal, RK20429). RT-qPCR was then conducted using Genious 2X SYBR Green Fast RT-qPCR Mix (Abclonal, RK21205). The expression of prognostic genes was normalized into GAPDH and then analyzed by $2^{-\Delta \Delta C_{t}}$ method. Primer sequences used in this analysis were displayed in Table 1.

**Table 1:** Primer sequences used for RT-qPCR.

Primer name	Sequences 5'–3'
GAPDH	F: 5′-AGGTCGGTGTGAACGGATTTG-3′
	R: 5′-TGTAGACCATGTAGTTGAGGTCA-3′
ZNF709	F: 5’-CATGACAATGCATCGGGCAG-3′
	R: 5′-TGTTGGAGCTTCTCACTATGCT-3′
ACSL6	F: 5’-GCCTGATGACCTCTCCATCG-3’
	R: 5’-GCCCACTGACTCTCTGTCAC-3’
FIP1L1	F: 5’-CGGAAACCTGGTGCTGATCT-3’
	R: 5’-GCTGTACCGTAATCTTGTTTGTGG-3’

### 2.13 Statistical analysis

Bioinformatic analysis was performed using R package (version 4.2.1) and python (version 3.11), and group difference was compared using Wilcoxon test. Experimental data were analyzed by GraphPad software (version 10.1.2), and group difference was compared through one-way ANOVA or t-test. Statistical significance was determined as *P* < 0.05.

## 3 Results

### 3.1 Astrocyte Cluster2 represented an astrocyte cluster with elevated ECM and PNN signatures in MDD

After QC, 229,256 cells (173,518 in MDD and 55,738 in control groups) and 43,208 genes remained (Supplementary Figure 1A). Top 2,000 HVGs were extracted (Supplementary Figure 1B), and 17,195 doublets were identified and 212,061 singlets were retained for further analysis (Supplementary Figure 1C). Subsequently, top 30 PCs were obtained (Supplementary Figure 1D), leading to the identification of 16 cell clusters (Figure 1A). According to marker genes (Figure 1B), seven cell types were annotated (Figure 1C), including oligodendrocytes, astrocytes, oligodendrocyte precursor cells (OPCs), microglia, endothelial cells, excitatory neurons (EN), inhibitory neurons (IN). The proportion analysis showed that EN exhibited more proportion in MDD and control samples (Figure 1D).

**Figure 1:**
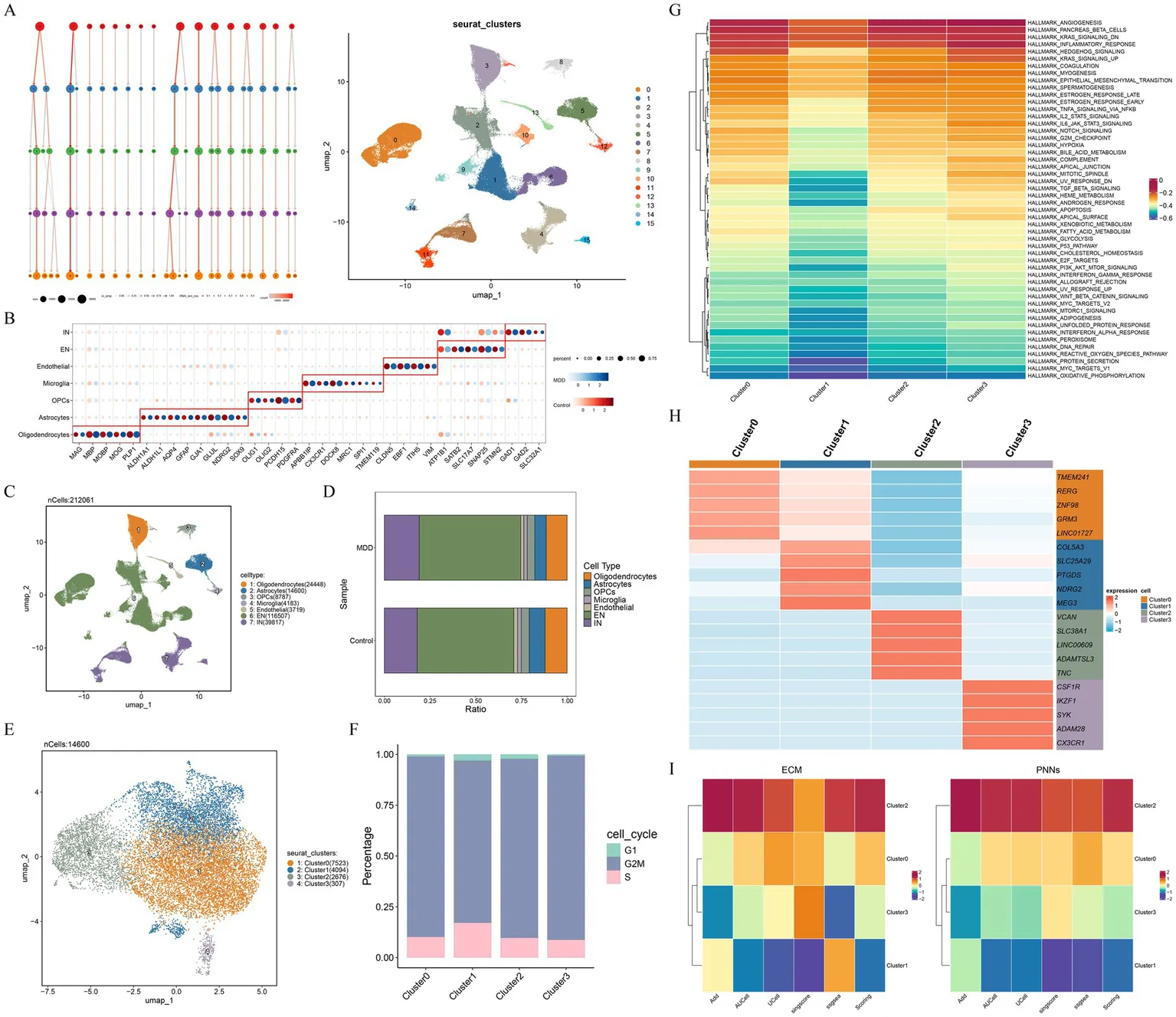
An astrocyte cluster (Cluster2) displays the highest ECM-PNN signature scores in major depressive disorder (MDD) single‑cell RNA sequencing (scRNA-seq) data. **(A)** Uniform Manifold Approximation and Projection (UMAP) visualization of 16 cell clusters. **(B)** The expression levels and percentage of canonical marker genes for each annotated cell type. **(C)** UMAP plot of seven annotated cell types. **(D)** The proportion of each cell type in MDD and control groups. **(E)** UMAP visualization of four clusters after reclustering of astrocytes. **(F)** Stacked bar plot showing cell cycle phase (G1, S, and G2M) distribution across astrocyte clusters. **(G)** Heatmap of 50 Hallmark pathway scores across astrocyte clusters. **(H)** Heatmap of top differentially expressed signature genes specific to each astrocyte cluster. **(I)** Heatmaps showing perineuronal nets (PNNs) and extracellular matrix (ECM) scores across astrocyte clusters using five integrated algorithms. The scRNA-seq datasets included GSE144136 (17 MDD and 17 control samples) and GSE213982 (20 MDD and 18 control samples).

Subsequently, owing to their association with PNNs and ECM (Tewari et al., 2024; Yang et al., 2024), reclustering of astrocytes was conducted and 4 cell clusters were identified (Figure 1E), including Cluster0, Cluster1, Cluster2, and Cluster3. Moreover, all cell subsets were predominantly composed of G2M-phase cells, and the Cluster1 exhibited a higher proportion of S-phase cells compared with other clusters (Figure 1F). Furthermore, four cell clusters displayed distinct gene-expression patterns, epithelial-mesenchymal transition, angiogenesis, inflammatory response were highly activated across all clusters (Figure 1G), and each cluster harbored its own signature genes (Figure 1H). For function enrichment, GO showed neuron-to-neuron synapse and postsynaptic density were shared by Cluster0, 1, and 2 in cellular component (CC). GTPase regulator activity and nucleoside-triphosphatase regulator activity were common to Cluster0 and 3 in molecular function (MF). KEGG revealed the IgSF CAM signaling pathway was commonly enriched in all four clusters, and Rap1 signaling pathway was significantly enriched in Cluster0, 1, and 3 (Supplementary Figures 2A–D). Collectively, these shared terms were closely implicated in the pathogenesis of MDD.

Finally, to evaluate the expression heterogeneity of PNNs and ECM signatures across four cell clusters, we adopted five algorithms. Consistent results were observed for both PNNs and ECM: Cluster2 showed overall high PNN and ECM signature scores and was selected for subsequent analyses (Figure 1I), regarded as key cell.

### 3.2 Biological pathways associated with astrocyte Cluster2

To ensure the network approximates a scale-free distribution, the soft threshold β was set to 8, and the mean connectivity approached 0 (Figure 2A). Subsequently, a co-expression network was constructed, and multiple co-expression modules exemplified were identified (Figure 2B). Module-trait correlation heatmap demonstrated that PNNs- and ECM-related gene sets were significantly associated with distinct modules across cell clusters. For instance, the PNNs gene set in Cluster1 showed strong significance with the blue module (*P* = 7.62 × 10^–13^; Figure 2C). Moreover, Pearson correlation analysis yielded three modules (brown, turquoise, and yellow) in Cluster2 showed the high correlation with PNNs and ECM (all cor > 0.3; Table 2). Thus, brown, turquoise, yellow modules were identified as key modules. The modules contained 114, 540, and 112 genes, totally, 766 key module genes.

**Figure 2:**
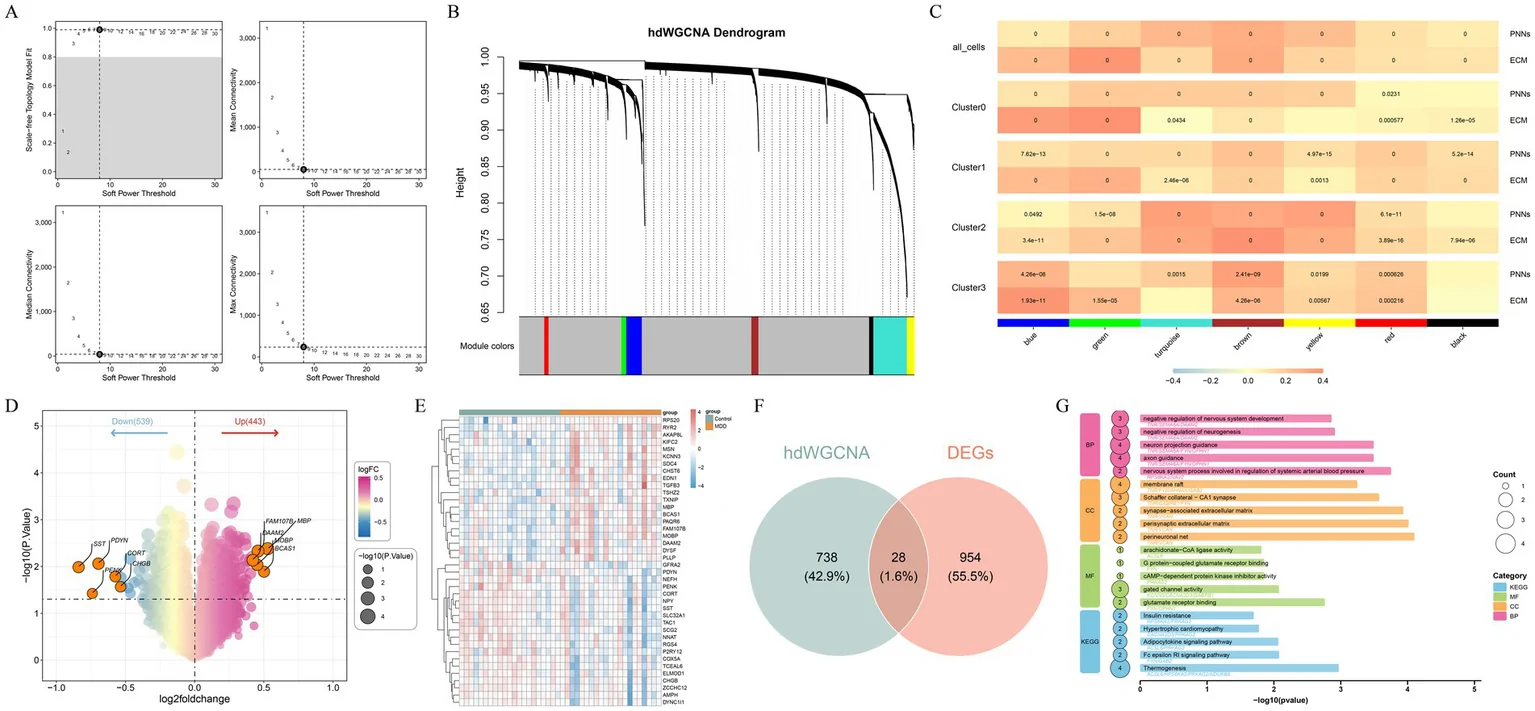
Biological pathway alterations associated with ECM/PNN-high astrocyte signatures **(A)** Determination of soft-thresholding power (*β*). **(B)** Dendrogram of co-expression modules identified by high-dimensional weighted gene co-expression network analysis (hdWGCNA). **(C)** Module-trait correlation heatmap. **(D)** Volcano plot of differentially expressed genes (DEGs) between MDD and control samples. **(E)** Heatmap of DEGs between MDD and control samples. **(F)** Venn diagram showing 28 intersection genes between key module genes and DEGs. **(G)** Gene Ontology (GO) and Kyoto Encyclopedia of Genes and Genomes (KEGG) enrichment analysis of 28 intersection genes.

**Table 2:** Pearson correlations between perineuronal nets (PNNs) and extracellular matrix (ECM) traits and gene modules in astrocyte Cluster2.

Gene module	ECM	PNNs
Cluster2.blue	0.1289	0.0386
Cluster2.green	0.2165	0.1100
Cluster2.turquoise	0.3215	0.3485
Cluster2.brown	0.4673	0.3153
Cluster2.yellow	0.3054	0.3455
Cluster2.red	0.1577	0.1270
Cluster2.black	0.0869	0.0301

Differential expression analysis identified 982 DEGs (443 upregulated and 539 downregulated) between control and MDD samples (Figures 2D,E).

Afterwards, the intersections of DEGs and key module genes were obtained, and 28 intersection genes were identified (Figure 2F). In addition, GO analysis was performed, and 546 terms were enriched including 379 biological process (BP, e.g., neuron projection guidance), 88 CC (e.g., synapse‑associated extracellular matrix) and 79 MF (e.g., glutamate receptor binding). These three terms converged on synapse assembly and neuronal remodeling. KEGG analysis enriched in 23 pathways (e.g., thermogenesis; Figure 2G), linking to these biological functions via calcium‑homeostasis‑mediated regulation.

### 3.3 Three biomarkers linked to astrocyte Cluster2

Two machine learning algorithms were employed to select feature genes. LASSO and SVM-RFE (minimum error rate = 0.183) respectively identified 13 and 24 feature genes (Figures 3A,B). The intersection of these feature genes yielded 13 common feature genes (Figure 3C). Additionally, three genes (ACSL6, FIP1L1, and ZNF709) were with the significant difference and consistent expression trends between MDD and control groups, which were identified as biomarkers. Among them, ACSL6 and ZNF709 were significantly down-regulated, whereas FIP1L1 was significantly up-regulated in MDD samples (Figure 3D). Moreover, the expression heterogeneity of biomarkers across cell types was performed. All biomarkers showed significant expression differences in oligodendrocytes, EN, and IN (Figure 3E).

**Figure 3:**
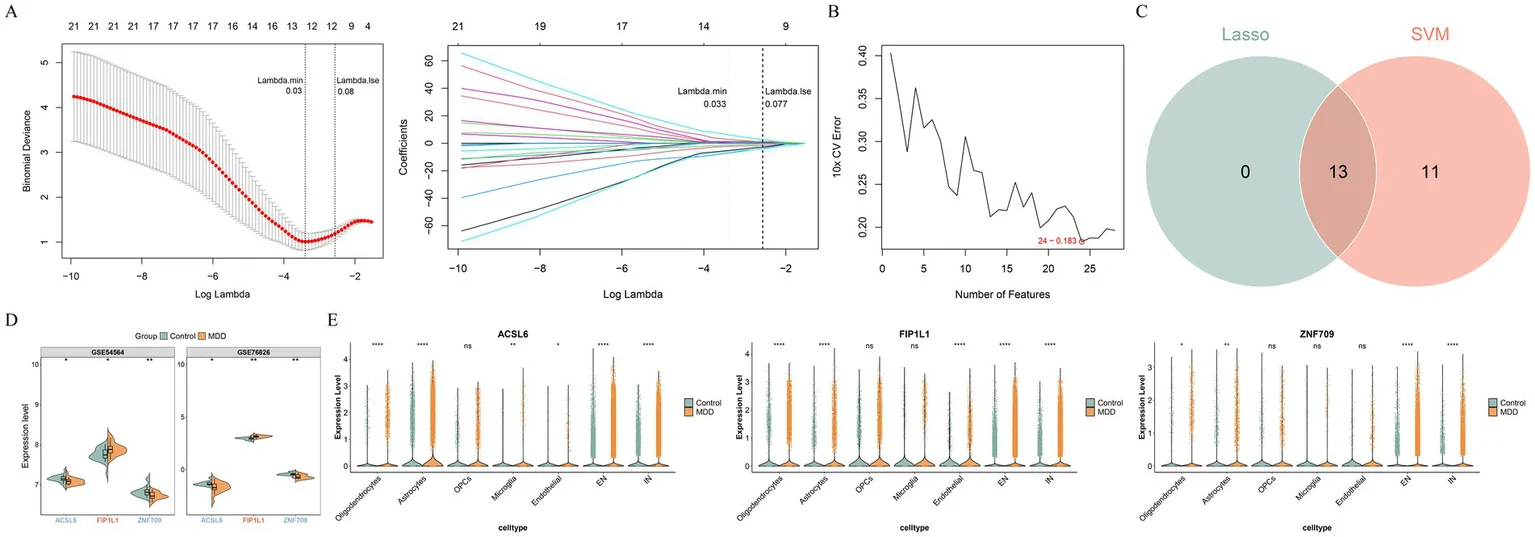
Expression patterns of three biomarkers linked to astrocyte cluster 2. **(A)** Least absolute shrinkage and selection operator (LASSO) regression coefficient paths for feature genes (right) and 10-fold cross-validation curve (left) with optimal *λE*. **(B)** Support vector machine-recursive feature elimination (SVM-RFE) feature selection showing error rate as a function of number of features. **(C)** Venn diagram showing 13 common feature genes identified by both LASSO and SVM-RFE. **(D)** Expression levels of ACSL6, FIP1L1, and ZNF709 in the training set (GSE54564; 21 MDD and 21 control samples) and validation set (GSE76826; 20 MDD and 12 control samples). **(E)** Violin plots showing expression heterogeneity of ACSL6, FIP1L1, and ZNF709 across seven annotated cell types. [In the figure, ns indicates no significant difference (*P* ≥ 0.05), while *, **, ***, and **** denote statistical significance at the levels of *P* < 0.05, *P* < 0.01, *P* < 0.001, and *P* < 0.0001, respectively.].

### 3.4 Dysregulated biological programs associated with ACSL6, FIP1L1, and ZNF709

To further assess the biological functions of biomarkers in MDD, GSEA was performed. Specifically, GSEA enrichment results showed that the biological processes enriched for ACSL6 were highly consistent between KEGG and Hallmark, mainly focusing on oxidative-stress response and energy-metabolism regulation (Figures 4A,B). For FIP1L1, the enriched biological processes in KEGG and Hallmark both pointed to cell-fate-related signal transduction, accompanied by negative enrichment of mitochondrial energy-metabolism-related pathways (Figures 4C,D). ZNF709 exhibited consistent negative enrichment of the NOTCH signaling pathway in both KEGG and Hallmark analyses; its KEGG enrichment was concentrated in amino-acid metabolism, sphingolipid metabolism and ribosome-related functions (Figures 4E,F). Collectively, GSEA results of all biomarkers demonstrated dysregulation of energy-metabolism-related pathways together with aberrant cell-fate-regulating signaling pathways.

**Figure 4:**
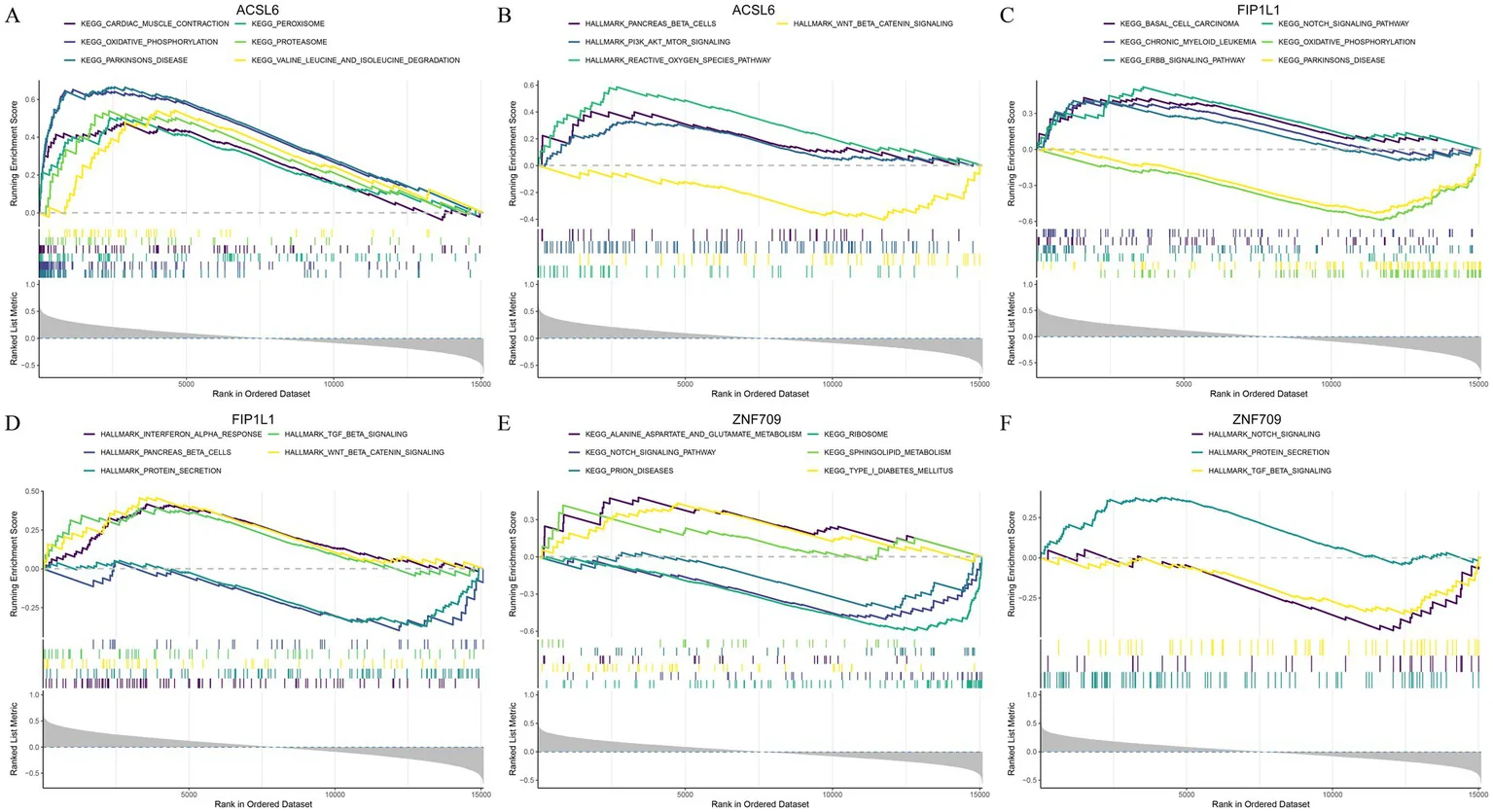
Diverse biological programs associated with ACSL6, FIP1L1, and ZNF709. **(A)** Gene set enrichment analysis (GSEA) results for ACSL6 in KEGG gene sets. **(B)** GSEA results for ACSL6 in Hallmark gene sets. **(C)** GSEA results for FIP1L1 in KEGG gene sets. **(D)** GSEA results for FIP1L1 in Hallmark gene sets. **(E)** GSEA results for ZNF709 in KEGG gene sets. **(F)** GSEA results for ZNF709 in Hallmark gene sets.

### 3.5 Altered cell‑cell communication and predicted functional outcomes of biomarker perturbation

Cell communication among cell types revealed that compared with the control group, the MDD group exhibited a larger number of cell‑cell interactions and elevated overall communication strength (Figure 5A). MDD group exhibited enhanced incoming and outgoing pathway signals (e.g., NRXN; Figure 5B). Pseudotime analysis inferred cell‑state trajectory along the 3‑2‑1 trajectory. The scores of ECM and PNNs signatures increased progressively over pseudotime. ACSL6, FIP1L1, and ZNF709 were highly expressed at trajectory terminus branch 1 (Figure 5C).

**Figure 5:**
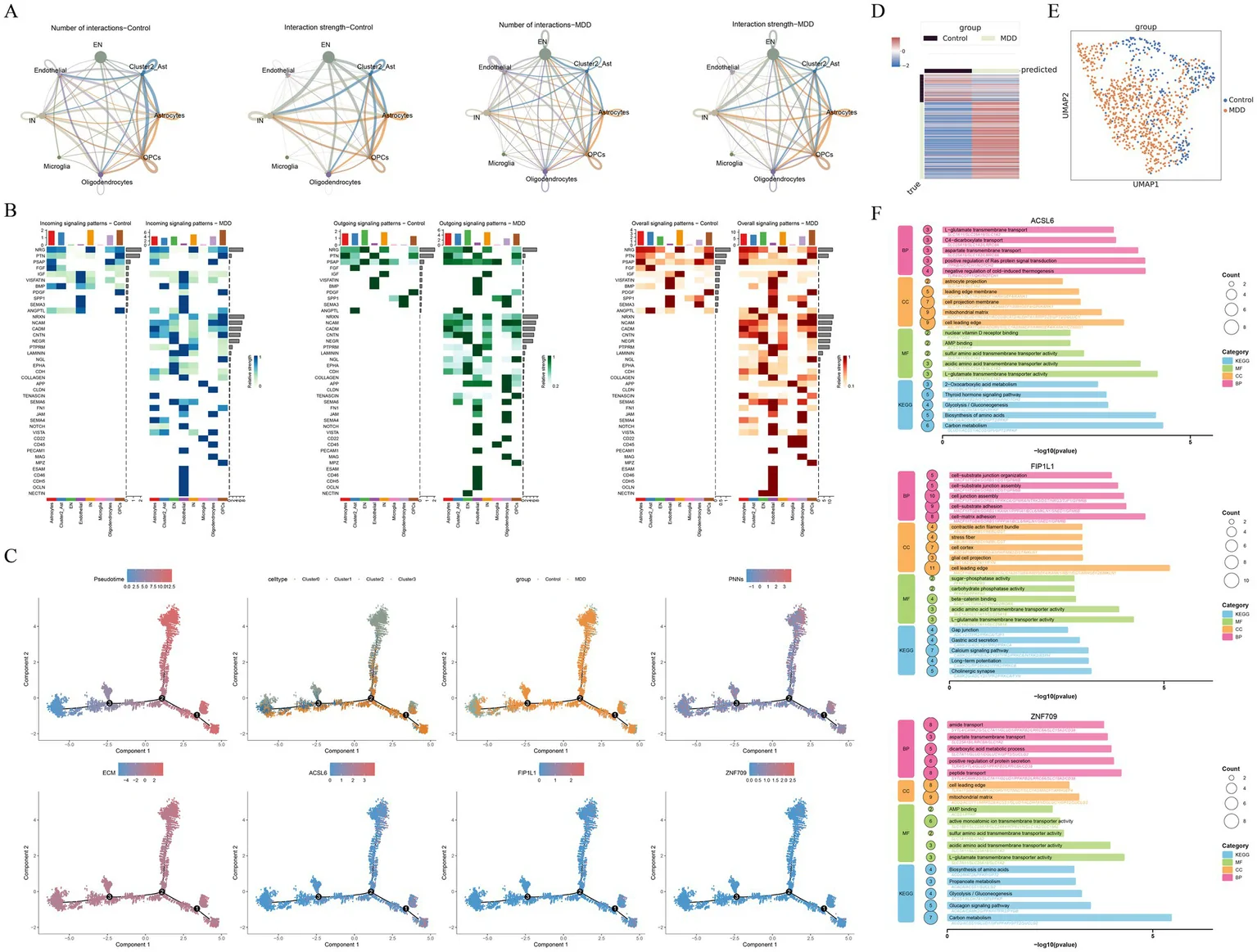
Altered intercellular signaling and predicted functional effects of biomarker perturbation for astrocyte cluster 2. **(A)** Cell-cell interaction networks in control and MDD groups showing number and strength of interactions among cell types. **(B)** Heatmap of signaling pathway outgoing/incoming signals in control and MDD groups. **(C)** Pseudotime trajectory showing developmental trajectory. **(D)** Heatmap of model outputs for MDD versus control classification. **(E)** UMAP visualization of fine-tuned Geneformer model predictions. **(F)** GO and KEGG enrichment analysis of top 100 perturbed genes following virtual perturbation of ACSL6, FIP1L1, and ZNF709.

Subsequently, model fine-tuning was performed on MDD and control astrocytes Cluster2.Model outputs and the distribution of cells by group were visualized in Figures 5D,E, respectively (Figures 5D,E). Afterwards, virtual knockout of biomarkers was performed via the fine-tuned model. We screened the top 100 perturbed genes upon knockout of biomarkers and performed functional enrichment analysis on them. Common enriched terms included cell leading edge (CC), L-glutamate and acidic amino-acid transmembrane transporter activity (MF). Cell adhesion, cytoskeleton- and synapse-related pathways were uniquely enriched after FIP1L1 knockout (Figure 5F). Consistent with previous GSEA results, these data suggest that the three biomarkers may be associated with amino-acid transport and energy metabolism-related programs, while FIP1L1 may additionally be linked to cell adhesion and synaptic signaling-related pathways. These outcomes represented in silico perturbation predictions and had not been validated by wet-lab experiments.

### 3.6 Stress‑responsive mRNA changes of biomarkers under glucocorticoid‑induced neuronal stress

To explore how ACSL6, ZNF709 and FIP1L1 change during CORT-triggered neuronal injury, we built an *in vitro* model. We first performed CCK-8 assays to pick a suitable modeling concentration. Treatment with 20 μM CORT for 24 h produced moderate neuronal damage without massive cell death and was adopted for subsequent experiments (Figure 6A). We then used RT-qPCR to measure biomarkers’ mRNA levels in control and model cells. Compared with control groups, the model group showed significantly reduced mRNA expression of ZNF709 (*P* < 0.01; Figure 6B) and ACSL6 (*P* < 0.001; Figure 6C), while FIP1L1 was markedly up-regulated (*P* < 0.01; Figure 6D). These data indicated that CORT-induced neuronal injury triggers obvious transcriptional shifts in ACSL6, ZNF709 and FIP1L1 in HT22 cells. Of note, these experiments were performed in neuronal HT22 cells and cannot directly validate astrocyte-specific function, ECM-PNN remodeling, or astrocyte-neuron crosstalk. They only provide evidence for stress-responsive transcriptional changes of these biomarkers.

**Figure 6:**
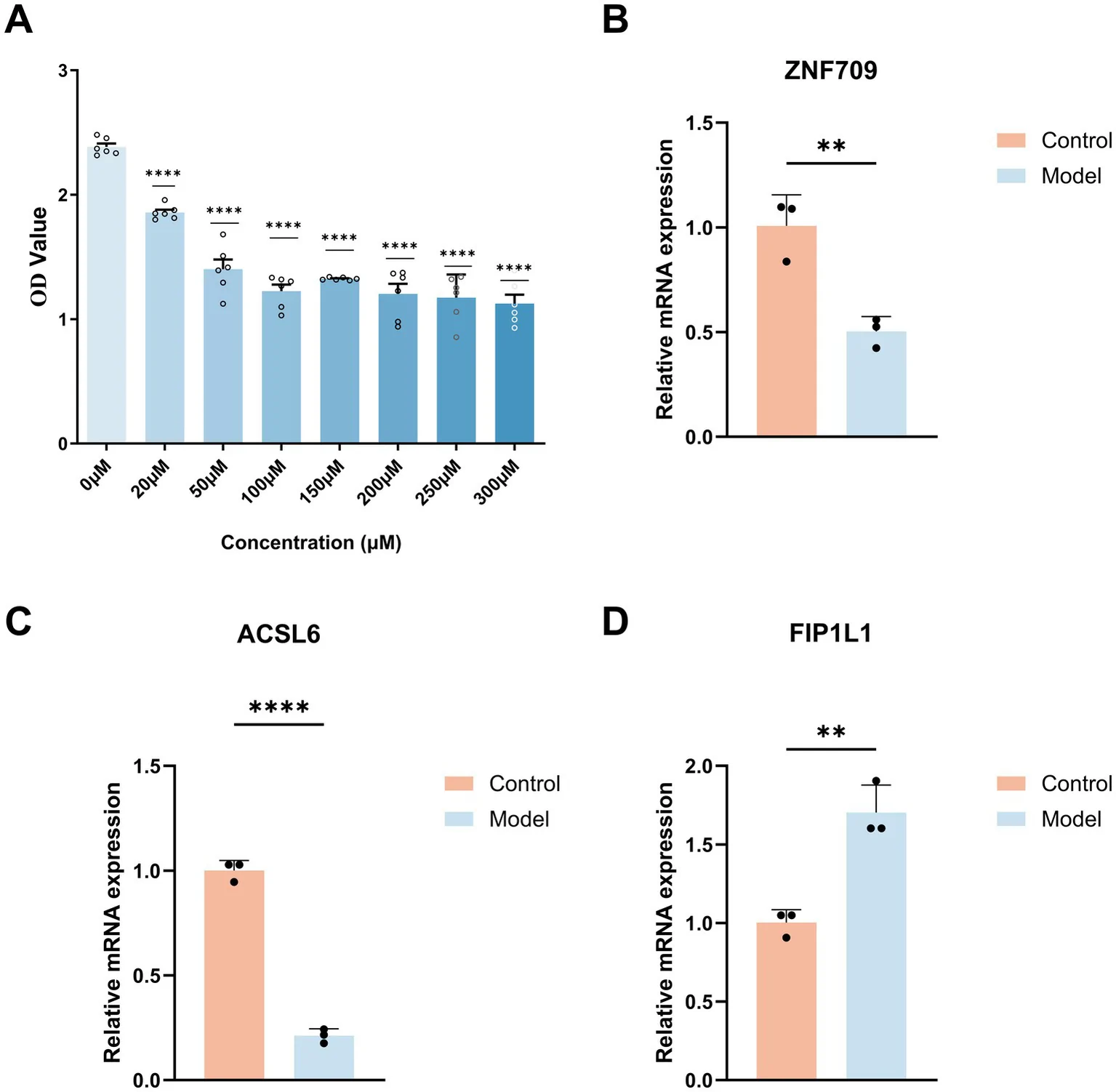
Stress‑responsive mRNA alterations of biomarkers under glucocorticoid‑induced neuronal stress. **(A)** Cell Counting Kit-8 (CCK-8) assay assessing HT22 cell viability following 24 h treatment with gradient concentrations of CORT. **(B)** RT-qPCR analysis of relative mRNA expression of ZNF709. **(C)** RT-qPCR analysis of relative mRNA expression of ACSL6. **(D)** RT-qPCR analysis of relative mRNA expression of FIP1L1. Data are presented as mean ± SD [*n* = 6 per concentration in **(A)**; *n* = 3 per group in **(B–D)**], and individual data points represent biological replicates. [In the figure, ns indicates no significant difference (*P* ≥ 0.05), while *, **, ***, and **** denote statistical significance at the levels of *P* < 0.05, *P* < 0.01, *P* < 0.001, and *P* < 0.0001, respectively.].

## 4 Discussion

### 4.1 Main findings

In this study, we proposed the following working model for MDD‑related ECM‑PNN disturbance: a distinct astrocyte cluster (Cluster2) characterized by elevated ECM-PNN signatures represents an ECM/PNN-associated transcriptional state in MDD, which may contribute to dysregulated extracellular homeostasis and aberrant astrocyte-neuron crosstalk. ACSL6, FIP1L1, and ZNF709 represented cross‑tissue molecular signatures correlated with this cell state. Of these biomarkers, FIP1L1 was of special interest because computational predictions linked it to post‑transcriptional control as well as cell‑adhesion and synaptic‑plasticity‑related programs.

### 4.2 Astrocyte Cluster 2 might contribute to aberrant neuroplasticity in MDD via ECM/PNN remodeling

In this study, astrocyte Cluster 2 exhibited overall high ECM and PNN signature scores across multiple scoring approaches, and was significantly enriched for functions such as synaptic regulation, cytoskeletal remodeling, ECM interactions, and signal transduction. Previous studies on postmortem brain tissue from MDD patients have observed reductions in GFAP-positive astrocytes in both the amygdala and hippocampus (Altshuler et al., 2010; Cobb et al., 2016); animal studies have also found that loss of prefrontal glial cells can induce depression-like behaviors (Banasr and Duman, 2008). These findings indicate that astrocyte abnormalities may contribute to the development and progression of MDD by affecting neurotransmitter clearance, synaptic support, and extracellular microenvironment homeostasis (Rajkowska and Stockmeier, 2013). This study pinpointed these abnormalities within a specific astrocyte cluster, suggesting that functional state shifts rather than astrocyte quantity changes may dominate MDD cellular pathology. Moreover, astrocytes are capable of synthesizing, assembling, and regulating various components of the brain ECM, and participate in the formation and dynamic remodeling of PNNs (Faissner et al., 2010; Simard et al., 2018). PNNs mainly surround parvalbumin-positive fast-spiking interneurons and regulate neuroplasticity via stabilizing synapses, tuning neuronal excitability and sustaining excitation-inhibition balance (Fawcett et al., 2019; Testa et al., 2019). Chronic stress can reprogram the transcriptional profile of astrocytes and upregulate the expression of molecules involved in ECM and PNN formation (Simard et al., 2018); in addition, the ECM component Neurocan is associated with stress susceptibility and the antidepressant effects of ketamine (Yu et al., 2022), and Semaphorin 3A–mediated PNN formation can also facilitate the persistence of depression-like behaviors by regulating the function of parvalbumin-positive interneurons (Mei et al., 2026). Combined with altered crosstalk between Cluster 2 and excitatory/inhibitory neurons and the rearrangement of ECM adhesion molecules, these results indicate that Cluster 2 may modulate synaptic stability, E/I balance and network plasticity via maintaining ECM/PNN homeostasis and glia-neuron crosstalk, thus driving MDD pathogenesis.

### 4.3 ACSL6, FIP1L1, and ZNF709 were determined as PNNs- and ECM- related biomarkers

In this study, three biomarkers were identified. Among them, ACSL6 and ZNF709 were downregulated in MDD, while FIP1L1 was upregulated. All three genes displayed concordant expression patterns in brain tissue and peripheral blood datasets, suggesting that they may represent cross-tissue molecular signatures associated with MDD-related alterations rather than changes restricted to a single biological compartment. The presence of similar expression trends across different tissues may indicate that these genes reflect broader disease-associated regulatory alterations in MDD. Given that MDD involves interactions among stress responses, metabolic disturbances, immune regulation, and neural dysfunction, cross-tissue molecular consistency may provide additional evidence for shared pathological processes. However, these findings do not imply that peripheral blood directly substitutes for brain-based molecular assessment, but rather suggest that these signatures may capture common biological changes associated with MDD. Long-chain acyl-CoA synthetase 6 (ACSL6) participates in the activation of polyunsaturated fatty acids and membrane phospholipid remodeling, and plays an important role in maintaining the enrichment of docosahexaenoic acid (DHA) and membrane lipid homeostasis in the brain (Fernandez et al., 2018). ACSL6 deficiency can lead to reduced DHA levels in the brain and altered membrane phospholipid composition, and is accompanied by changes in synapse-related gene expression, cognitive function, and neuroinflammation (Fernandez et al., 2021). Therefore, the downregulation of ACSL6 in this study may reflect abnormalities in lipid metabolism and cell membrane homeostasis in the brains of individuals with MDD. Membrane lipids regulate receptor localization, adhesion and cytoskeleton dynamics. Reduced ACSL6 may impair ECM signal perception, connecting lipid metabolic disturbance with ECM/PNN remodeling. Factor interacting with PAPOLA and CPSF1 (FIP1L1) is an important component of the cleavage and polyadenylation specificity factor complex, capable of binding uridine-rich RNA sequences and participating in mRNA 3′-end processing (Kaufmann et al., 2004). FIP1L1 can also regulate alternative polyadenylation site selection, thereby influencing transcript stability, localization, and translation efficiency (Lackford et al., 2014). Unlike single-target regulation, altered FIP1L1 expression globally remodels post-transcriptional processing, modulating cell adhesion, cytoskeleton dynamics, transport and secretion. This study identified elevated FIP1L1 in MDD brain and peripheral blood, implying it bridges aberrant RNA processing, astrocyte state shifts and ECM/PNN remodeling. Zinc finger protein 709 (ZNF709) belongs to the KRAB zinc finger protein family, an important group of transcriptional regulators in higher vertebrates that can participate in gene expression and epigenetic regulation by recruiting corepressor complexes (Ecco et al., 2017). Related studies have shown that mouse ZFP871 (a homolog of human ZNF709) can modulate p53 stability and the cellular stress response (Yan et al., 2016). In this study, ZNF709 showed sustained downregulation, suggesting that MDD may be accompanied by alterations in specific programs of transcriptional repression and cellular stress regulation. Overall, ACSL6, FIP1L1, and ZNF709 participate in membrane lipid metabolism, RNA processing and transcriptional regulation, respectively. This indicates MDD-related ECM/PNN dysfunction arises from coordinated disruption of lipid homeostasis, post-transcriptional and transcriptional control, rather than a single pathway. Concordant expression of the three genes in brain and peripheral blood supports their potential as candidate biomarkers. Notably, FIP1L1 was persistently upregulated in both tissues. Enrichment analysis linked FIP1L1 to WNT/β-catenin, TGF-β and interferon-α signaling, marking it as a key target for subsequent virtual perturbation analyses. To further validate these observations, we used CORT-treated HT22 cells to recapitulate glucocorticoid-driven neuronal injury, a widely-used *in vitro* cell model for depression-associated stress. RT-qPCR showed that CORT exposure lowered ACSL6 and ZNF709 mRNA while increasing FIP1L1 transcript abundance. These expression patterns matched those seen in brain tissue and peripheral blood datasets. Such consistent results across different sample sources support the reliability of these biomarkers.

### 4.4 FIP1L1 might remodel astrocyte–ECM/PNN–neuron interactions via posttranscriptional regulation

Virtual knockout of three biomarkers uncovered shared enrichment in amino acid transport and energy metabolism. While perturbation of ACSL6 and ZNF709 only altered metabolic and amino acid transport programs, uniquely, FIP1L1 perturbation additionally enriched pathways related to cell adhesion, cytoskeleton dynamics and synaptic signaling, consistent with preceding GSEA results. These Geneformer-based perturbation results should be interpreted as computational predictions of potential downstream transcriptional changes rather than direct experimental validation of gene function. Nevertheless, the predicted pathways showed consistency with previous GSEA findings and reported biological functions of the candidate genes. For example, the predicted association of FIP1L1 with cell adhesion, cytoskeletal organization, and synaptic-related pathways is compatible with its known role in RNA processing and transcript regulation, supporting its potential involvement in broader cellular state remodeling. Biologically, this finding is credible. FIP1L1 governs mRNA 3’-end processing and alternative polyadenylation; its expression shifts simultaneously modulate transcript stability, localization and translation (Davis et al., 2022). Therefore, FIP1L1 abnormalities may not act only on a few effector genes, but rather exert broad post-transcriptional regulation.

GSEA revealed FIP1L1-related pathways associated with neuroplasticity, stress resilience, inflammation and antidepressant responses (Dias et al., 2014; Pilar-Cúellar et al., 2014; Caraci et al., 2018), with IFN-α driving synaptic and neuroendocrine dysfunction in depression (Hoyo-Becerra et al., 2014). Consistent omics and perturbation data support FIP1L1 as a bridge linking abnormal RNA processing, stress immunity and neuroplastic remodeling. In addition, cell communication analysis identified disrupted ligand-receptor signaling between astrocyte Cluster 2 and neurons, impairing adhesion, ECM interaction and intercellular transmission. Prior MDD studies reported abnormal glutamate/GABA signaling (Choudary et al., 2005), reduced astrocytic glutamate transporters (Chandley et al., 2013) and stress-related astrocytic and gap junction defects (Aten et al., 2023). The Cx43 glial network connects glutamate balance and depressive pathology (Zhang et al., 2022), supporting our results. Collectively, elevated FIP1L1 may disrupts astrocytic post-transcriptional regulation, disturbs ECM/PNN homeostasis and astrocyte-neuron crosstalk, and impairs synaptic plasticity in MDD, offering directions for further FIP1L1-related research. Our *in vitro* data lend further support to this idea. FIP1L1 was upregulated under CORT-mediated stress, consistent with its known role in RNA processing, which may in turn modify adhesion-related molecules and synaptic components. Therefore, the HT22 stress model was used to provide complementary evidence that the identified candidate genes retain stress-responsive transcriptional regulation under an independent neuronal stress condition, rather than to directly validate astrocyte-specific ECM/PNN remodeling.

### 4.5 Innovations and limitations

This study has three core innovations. First, gene scoring and hdWGCNA mapped ECM/PNN signatures to specific astrocyte clusters, revealing the single‑cell basis of extracellular remodeling in MDD. Second, we identified cross‑tissue biomarkers associated with this disease‑relevant astrocyte state. Third, we included experimental validation using the CORT‑triggered neuronal‑injury model. This brings in a translational perspective and confirms that these biomarkers can respond to stress‑related stimuli. However, this work also bears limitations. All data originate from public cross‑sectional datasets with potential technical and clinical heterogeneity. Computational predictions lack experimental confirmation. Our *in vitro* validation was performed in neuronal HT22 cells and cannot directly verify astrocyte‑specific ECM‑PNN remodeling. Independent cohorts, astrocyte-based functional experiments and *in-vivo* animal models are needed to validate the identified astrocyte states, biomarkers and ECM‑PNN‑related mechanistic hypotheses.

## 5 Conclusion

This study identified an ECM-PNN-high astrocyte transcriptional state associated with MDD. Three cross‑tissue biomarkers (ACSL6, FIP1L1, and ZNF709) linked to this astrocyte state were characterized. Computational analyses suggest that these genes were involved in metabolic processes, while FIP1L1 additionally exhibited predicted associations with cell‑adhesion and synaptic‑plasticity‑related programs. Experiments in a glucocorticoid‑induced neuronal stress paradigm confirmed stress‑responsive mRNA expression changes of these three biomarkers. Collectively, our findings proposed a biologically plausible working model linking astrocyte ECM‑PNN remodeling to MDD pathogenesis. Further functional *in vitro* and *in vivo* experiments were required to validate this mechanistic hypothesis.

## Data Availability

The datasets presented in this study can be found in online repositories. The names of the repository/repositories and accession number(s) can be found in the article/Supplementary material.

## Footnotes

1
http://www.ncbi.nlm.nih.gov/geo/

2
https://www.gsea-msigdb.org/gsea/msigdb

3
https://matrisomedb.org/

4
https://igordot.github.io/msigdbr/
