# Supplementary material for: Integrative transcriptomics reveals an ECM/PNN-high astrocyte state and cross-tissue biomarkers in major depressive disorder
Source: Front Mol Neurosci. 2026 Oct 23;19:1995597. doi: 10.3389/fnmol.2026.1995597 (PMC13752861; doi:10.3389/fnmol.2026.1995597)
Supplement: Supplementary file 1 [file Data_Sheet_1.docx]

Supplementary Material

# 1 Supplementary Data

**Drug prediction and molecular docking**

To identify drugs targeting biomarkers, related drugs from the DSigDB (https://dsigdb.tanlab.org/DSigDBv1.0/) database were retrieved, and a drug-biomarker interaction network was visualized. Biomarkers and drugs with suitable structures available in PubChem (https://pubchem.ncbi.nlm.nih.gov/) database were selected for molecular docking. The 3D protein structures of biomarkers were downloaded in protein data bank (PDB) format from the UniProt (https://www.uniprot.org) database. Drug 3D structures were obtained from PubChem database. Finally, molecular docking was performed in gnina (version 1.1.0) (McNutt et al., 2021) to evaluate binding free energy. Docking complexes were visualized using PyMOL (version 2.6.0) (Seeliger and de Groot, 2010).

**Results**

**Drug prediction and molecular docking**

To analyze the interactions between biomarkers and their corresponding drugs, a drug-biomarker network was built comprising 26 drugs (e.g., trichostatin A-ZNF709, theophylline-FIP1L1 and quercetin-ACSL6) (Figure S3A). Molecular docking was performed to evaluate binding affinity. Among them, quercetin exhibited the strongest binding affinity (-9.75 kcal/mol) toward ACSL6, while theophylline showed weak binding (-4.85 kcal/mol) toward FIP1L1 (Table S3). Their complexes’ molecular docking were visualized in Figure S3B.

**Reference**

McNutt, A.T., Francoeur, P., Aggarwal, R., Masuda, T., Meli, R., Ragoza, M., et al. (2021). GNINA 1.0: molecular docking with deep learning. *J Cheminform* 13(1)**,** 43. doi: 10.1186/s13321-021-00522-2.

Seeliger, D., and de Groot, B.L. (2010). Ligand docking and binding site analysis with PyMOL and Autodock/Vina. *J Comput Aided Mol Des* 24(5)**,** 417-422. doi: 10.1007/s10822-010-9352-6.

# 2 Supplementary Figures and Tables

**Table S1 Molecular docking binding free energies of biomarkers with predicted drugs**.

| Gene | UniProt | PDB | Targeted drug | Drug CID | Molecular binding energy | Number of hydrogen bonds |
| --- | --- | --- | --- | --- | --- | --- |
| ZNF709 | Q8N972 | AF-Q8N972-F1 | trichostatin A | CID444732 | -6.39 | 2 |
| FIP1L1 | Q6UN15 | AF-Q6UN15-F1 | theophylline | CID2153 | -4.85 | 5 |
| ACSL6 | Q9UKU0 | AF-Q9UKU0-F1 | quercetin | CID5280343 | -9.75 | 4 |

Note. UniProt, Universal Protein Resource; PDB, Protein Data Bank; CID, Compound Identifier. Binding energy is given in kcal/mol.

## 2.1 Supplementary Figures

**Supplementary Figure 1 Quality control of single-cell RNA sequencing (scRNA-seq) data. (A)** Quality control. **(B)** Top 2,000 highly variable genes identified by vst method. **(C)** UMAP visualization and proportion of doublet identification. **(D)** Top 30 principal components selected for downstream analysis.

**Supplementary Figure 2 Functional enrichment analysis of marker genes in astrocyte clusters. (A)** GO and KEGG enrichment analysis of marker genes in Cluster0. **(B)** GO and KEGG enrichment analysis of marker genes in Cluster1. **(C)** GO and KEGG enrichment analysis of marker genes in Cluster2. **(D)** GO and KEGG enrichment analysis of marker genes in Cluster3.

**Supplementary Figure 3** Drug prediction and molecular docking. (A) Drug-biomarker interaction network. (B) Molecular docking complexes.
